# Homologous platelet gel on radiation-induced dermatitis in a patient receiving head and neck radiotherapy plus cetuximab: A case report

**DOI:** 10.1097/MD.0000000000034779

**Published:** 2023-08-25

**Authors:** Monica Guberti, Davide Schiroli, Chiara Marraccini, Genny Mazza, Cinzia Iotti, Roberto Baricchi, Barbara Iotti, Lucia Merolle

**Affiliations:** a Nursing Research and EBP Unit – Health Professions Department, Azienda USL-IRCCS di Reggio Emilia, Reggio Emilia, Italy; b Transfusion Medicine Unit, Azienda USL-IRCCS di Reggio Emilia, Reggio Emilia, Italy; c Oncology Day Care Unit, Azienda USL-IRCCS di Reggio Emilia, Reggio Emilia, Italy; d Radiation Oncology Unit, Azienda USL-IRCCS di Reggio Emilia, Reggio Emilia, Italy.

**Keywords:** head, neck cancer, platelet gel, radiodermatitis

## Abstract

**Introduction:**

Acute radiodermatitis is a significant complication of cancer radiotherapy, and platelet-based therapies are emerging as potential new treatments.

**Main symptoms and important clinical findings:**

In this report, we present the case of a patient with head and neck cancer undergoing radiotherapy combined with the monoclonal antibody cetuximab. After 4 weeks of this treatment, the patient developed cutaneous radiation dermatitis. Despite receiving standard treatment with corticosteroids and emollient cream, the lesion did not improve.

**Main diagnosis:**

cutaneous radiation dermatitis on head and neck cancer patient.

**Therapeutic interventions:**

Topical application of platelet gel was initiated on the wound. From the second week of radiotherapy to the 4th week, homologous platelet-rich plasma was applied on the dermatitis using a bandage, 4 times a day.

**Outcomes:**

The topical treatment with homologous platelet gel resulted in complete healing of the radiodermatitis, including restoration of the epidermis, reepithelialization, and reduction in associated pain.

**Conclusion:**

homologous platelet gel might be an alternative to standard treatment of radiation dermatitis.

## 1. Introduction

Radiation therapy is a commonly used treatment modality for various types of cancer.^[1]^ However, a significant side effect of radiotherapy, chemotherapy, or epidermal growth factor receptor inhibitors is the development of acute radiodermatitis, which can occur after as few as 10 to 15 sessions and affects approximately 95% of patients with head and neck tumors.^[2]^ The symptoms of acute radiodermatitis are categorized into 3 levels: grade 1 (mild erythema), grade 2 (dry desquamation), and grade 3 (severe moist desquamation). These symptoms significantly impact the quality of life for affected patients, often causing pain, ulceration, swelling, increased infection risk, and reduced adherence to the treatment protocol.^[3,4]^

Conventional treatments for acute radiation-induced injuries typically involve topical application of antibacterial creams, corticosteroids, anesthetics, hydrogels, or systemic antibiotics in cases of infection.^[1,4]^ Some literature reports evidence-based cases of effective remedies and non-pharmaceutical agents.^[4–6]^ However, these treatments have limited efficacy in promoting reepithelialization of the skin compromised by radiotherapy. reepithelialization is a well-known process facilitated by the proliferation and migration of keratinocytes and fibroblasts. Platelets play a crucial role in this process by releasing bioactive molecules such as growth factors and cytokines, which possess chemotactic and mitogenic properties that modulate the repair mechanisms of injured skin.^[7–11]^ In line with the biostimulatory role of platelets, several studies have documented the healing properties of platelet concentrates in the treatment of radiodermatitis lesions.^[12–17]^

In particular, the use of platelet gel has emerged as a promising new treatment modality for radiodermatitis. This approach involves the utilization of platelet-rich plasma (PRP), a concentrated source of platelets that can be activated by exposure to calcium ions and the addition of thrombin. This activation transforms PRP into a gel-like substance that is both pliable and adhesive, making it suitable for local treatment of damaged tissues.^[7]^ While there have been few reported clinical cases of acute or chronic radiodermatitis treated with platelet gel, either alone or in combination with other therapies, most cases have demonstrated complete recovery of the affected tissue.^[12]^

Furthermore, to the best of our knowledge, the use of homologous or allogeneic sources to produce PRP has never been tested for these types of lesions. Herein, we present the case of a patient with radiodermatitis treated exclusively with topical application of homologous platelet gel, which resulted in complete healing.

## 2. Methodological aspects

**Figure 1.**
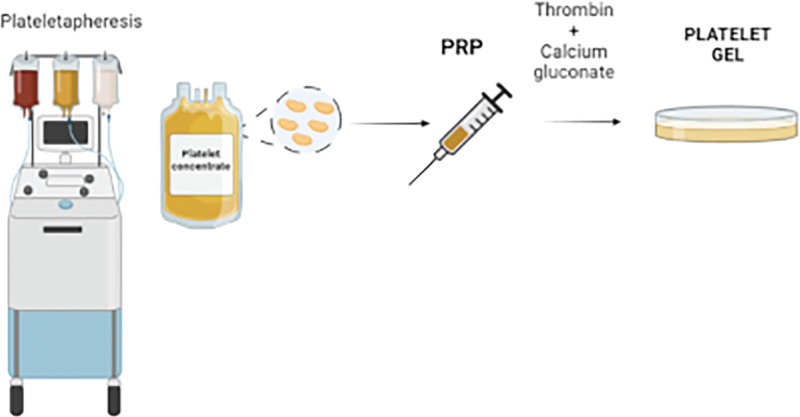
Scheme of platelet gel preparation. The PRP unit, obtained by plateletapheresis, is combined with calcium gluconate 1000 mg/10 mL and thrombin. This process permits the complete platelet activation and lead to the platelet gel formation which can be then applied on injured epidermis as bio-film. Image created with BioRender.com. PRP = platelet-rich plasma.

Homologous PRP was collected at Casa del Dono di Reggio Emilia by apheresis procedure from 1 single donor. The PRP was used to reconstitute platelet gel: briefly, 8 mL syringe of platelet-rich plasma was mixed with 1mL of calcium gluconate 1000 mg/10 mL (Bioindustria L.I.M.m Novi Ligure, Italy) and 2 mL of thrombin in order to achieve complete platelets activation and coagulum formation (Fig. 1). PRP and Thrombin are byproduct of blood routinely obtained at Transfusion Medicine Unit of the AUSL-IRCCS di Reggio Emilia.^[18]^ Medication was performed at home by expert nurse: the skin was cleansed with Amukine Med 0.05 % (Angelini, Rome, Italy), NaCl 0.9% (Fresenius, Modena, Italy) and dried with sterile gauze. Thereafter, platelet gel was applied and the lesions covered with a transparent dressing (Tegaderm^3M^) for 24 hours.

The radiation oncology/toxicity grading scale^[19]^ and the common terminology criteria for adverse events score v4.03 (CTCAE v 4.03) score,^[20]^ were used to assess the grade of dermatitis. The numeric rating scale (NRS) from 0 (no pain) to 10 (worst pain) was used to assess pain.

Specific ethical approval was not required for the conduction of this case study. Data are presented without revealing the patient’s identity and we acquired both the patient’s and the legal guardian’s informed consent for the publication of the anonymised data.

## 3. Case presentation

The patient is a 78-year-old man diagnosed in 2019 with squamous cell carcinoma of the oropharynx p16 + (left tonsil) in stage cT2 N1. Comorbidities included hypertension, in treatment with Ramipril 5 mg.

In December 2019, the patient underwent curative radiation treatment in combination with cCetuximab. The radiotherapy was administered using Tomotherapy HiART (Accuray Inc) with a total dose of 66 Gy delivered in 30 daily fractions over 5 days per week. Cetuximab was administered with a loading dose of 400 mg/m² 1 week prior to radiation therapy, followed by a weekly dose of 250 mg/m² during the 6-week course of radiation. Premedication with chlorphenamine (10 mg) and dexamethasone (8 mg) was consistently given 1 hour before Cetuximab administration to minimize allergic reactions.

**Figure 2.**
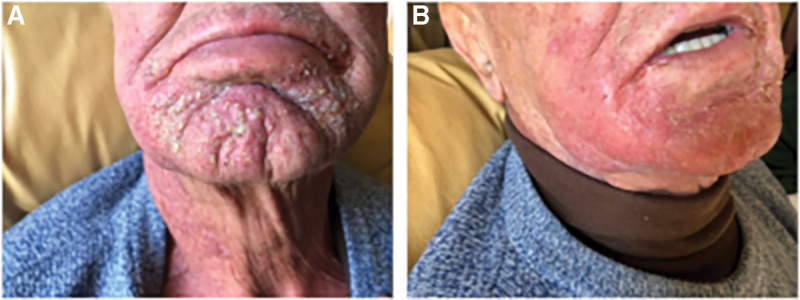
Condition of the skin after 4^th^ weeks of radiotherapy (A) and immediately after the first application of platelet gel derived from apheresis (B). In the first image (A) it is clearly visible a radiodermatitis area characterized by moist desquamation with tender, red skin associated with little hemorrhagic crusting and erythema atrophic plaques (RTOG and CTCAE v 4.03 score 2), (B) first application of platelet gel, improved radiodermitis erythema and cutaneous trophism, see the absence of erythema plaques (RTOG and CTCAE v 4.03 score 1). CTCAE = common terminology criteria for adverse events score, RTOG = radiation oncology/toxicity grading scale.

After 4 weeks of radiotherapy, the patient developed grade 2 radiodermatitis on the chin and part of the cheeks (Fig. 2A). The affected area exhibited extreme dryness, flaking, brisk erythema, and edema of the skin. Over time, it progressed to moist desquamation, with tender and red skin, atrophic erythematous plaques in non-folded areas, and bleeding with hemorrhagic crusting upon minor trauma (data not shown). Grade 2 mucositis was also present, causing severe pain (NRS 9), odynophagia, and feeding difficulties.

The patient was then initiated on daily oral corticosteroid therapy (dexamethasone 4 mg) and received analgesic treatment with opioids (Fentanest 25 μg/day) via parenteral administration. Before initiating platelet gel therapy, a topical skin treatment with a soothing cream (Crema Lenitiva MOST) was applied to the irradiated skin 2 to 3 times a day, excluding radiotherapy sessions. However, the radiodermatitis did not improve, and the patient continued to experience pain.

Topical administration of platelet gel commenced at the end of the 4th week of radiotherapy and continued until the end of the 5th week. The treatment was performed twice daily on radiotherapy-free days, totaling 4 applications. The platelet gel was always covered with a Tegaderm 3M Transparent dressing and left in place for 24 hours. After the first application, the patient’s erythema improved, and skin trophism showed noticeable enhancement (Fig. 2B). Despite maintaining both chemotherapy and radiotherapy regimens, at the end of the platelet gel treatment cycle, the area had completely reepithelialized, and the patient no longer experienced pain. No side effects were observed during or after treatment, and the treatment was well tolerated. The pain, assessed using the NRS grading system, reduced to grade 0 (NRS 0), and both radiation oncology/toxicity grading scale and CTCAE v 4.03 scores were rated as 1.

## 4. Discussion and conclusion

In this case report, we present the clinical history of a 78-year-old man who achieved complete healing of radiation-induced skin injury (radiodermatitis) following treatment with homologous platelet gel. Similar positive outcomes have been observed in previous studies, such as the 1 conducted by Iervolino et al,^[12]^ which reported the use of autologous platelet gel in a group of 10 patients with third and 4th degree acute and chronic radiodermatitis.

In our case, the progression of the skin lesion strongly suggests the significant efficacy of platelet gel in treating radiodermatitis. We observed the resolution of the injury, and the patient reported complete remission of severe pain symptoms after 2 weeks of gel application, despite concurrent radiotherapy and chemotherapy. Platelet gel was found to be easy to use and effective in reducing skin damage, which had previously been unresponsive to other therapeutic approaches. The dressing was performed at home, eliminating the need for hospitalization, and the gel itself was prepared by the patient care giver at home.

As widely reported, radiation therapy-induced damage can impair the healing process mediated by growth factors, leading to dermal atrophy.^[6]^ The healing efficacy of platelet gel lies in its high content of growth factors released from platelet α-granules upon activation, which are responsible for the regeneration of the skin.^[7–11]^ Moreover, the use of homologous PRP obtained through apheresis as the starting material for platelet gel production has proven to be safe and devoid of side effects. It has the added advantage of being readily available at blood transfusion centers and offering greater standardization compared to other similar products, such as autologous PRP. It should be noted that the effectiveness of platelet-based therapies heavily depends on the concentration of bioactive molecules released by platelets, which is influenced by the preparation method. The wide heterogeneity of protocols for autologous PRP production reduces the reproducibility of PRP composition,^[8,10,11]^ potentially affecting its clinical efficacy.

Further studies are needed to establish standardized methods for the application and formulation of platelet gel in the treatment of radiation-induced dermatitis. Improvements in the treatment regimen and application technique are also necessary to enhance patient quality of life. One aspect that should not be overlooked in platelet-based therapies is their oncogenic potential. Molecules and growth factors contained in platelet α-granules, such as vascular endothelial growth factor, platelet-derived growth factor, epidermal growth factor receptor, HER, and transforming growth factor-beta, are involved in interactions that contribute to tumor cell immortality, including angiogenesis, lymphangiogenesis, proliferation, defective differentiation, and inhibition of apoptosis.^[7,9,21]^ While the hypothesis of PRP-related malignant transformation has not been confirmed, it is crucial to encourage research activities that thoroughly investigate platelet interactions with the surrounding injection or application site, in order to eliminate any potential risk of recurrence in patients who have undergone complete tumor excision.

The results presented here provide initial evidence for the development of clinical studies aimed at evaluating the effects of homologous PRP on patients undergoing radiotherapy, with the goal of preventing or treating acute radiation-induced dermatitis.

## Acknowledgements

This study was partially supported by Italian Ministry of Health—Ricerca Corrente Annual Program 2024. No funding that could be perceived as a conflict of interest was received for this study. Authors want to acknowledge the nurses and medical staff of Radiotherapy, Oncology and Transfusion Medicine Unit.

## Author contributions

**Conceptualization:** Monica Guberti, Davide Schiroli, Barbara Iotti, Lucia Merolle.

**Data curation:** Monica Guberti, Davide Schiroli, Lucia Merolle.

**Investigation:** Monica Guberti, Genny Mazza, Cinzia Iotti.

**Methodology:** Monica Guberti, Davide Schiroli, Barbara Iotti, Lucia Merolle.

**Supervision:** Davide Schiroli, Chiara Marraccini, Lucia Merolle.

**Writing – original draft:** Monica Guberti, Davide Schiroli, Lucia Merolle.

**Writing – review & editing:** Davide Schiroli, Chiara Marraccini, Genny Mazza, Roberto Baricchi, Lucia Merolle.

## References

[1] Seité S Bensadoun RJ Mazer JM. Prevention and treatment of acute and chronic radiodermatitis. Breast Cancer (Dove Med Press). 2017;9:551–7. 29138594 10.2147/BCTT.S149752 PMC5677297

[2] Presta G Puliatti A Bonetti L. Effectiveness of hyaluronic acid gel (Jalosome soothing gel) for the treatment of radiodermatitis in a patient receiving head and neck radiotherapy associated with cetuximab: a case report and review. Int Wound J. 2019;16:1433–9. 31475472 10.1111/iwj.13210 PMC7948705

[3] Classification of dermatitis associated with Bioradiotherapy (BRT) concomitant with Cetuximab (CTX) (Russi, 2013).

[4] Iacovelli NA Torrente Y Ciuffreda A. Topical treatment of radiation-induced dermatitis: current issues and potential solutions. Drugs Context. 2020;9:1–13. 10.7573/dic.2020-4-7 PMC7295106 32587626

[5] Russi EG Moretto F Rampino M. Acute skin toxicity management in head and neck cancer patients treated with radiotherapy and chemotherapy or EGFR inhibitors: literature review and consensus. Crit Rev Oncol Hematol. 2015;96:167–82. 26187236 10.1016/j.critrevonc.2015.06.001

[6] Kong M Hong SE. Topical use of recombinant human epidermal growth factor (EGF)-based cream to prevent radiation dermatitis in breast cancer patients: a single-blind randomized preliminary study. Asian Pac J Cancer Prev. 2013;14:4859–64. 24083759 10.7314/apjcp.2013.14.8.4859

[7] Oneto P Julia E. PRP in wound healing applications. Platelets. 2021;32:189–99. 33251921 10.1080/09537104.2020.1849605

[8] Pulcini S Merolle L Marraccini C. Apheresis platelet rich-plasma for regenerative medicine: an in vitro study on osteogenic potential. Int J Mol Sci. 2021;22:8764. 34445472 10.3390/ijms22168764 PMC8395746

[9] Bhatnagar P Xian Law J Shiow-Fern N. Delivery systems for platelet derived growth factors in wound healing: a review of recent developments and global patent landscape. J Drug Delivery Sci Technol. 2022;71:103270.

[10] Mazzucco L Balbo V Cattana E. Not every PRP-gel is born equal. Evaluation of growth factor availability for tissues through four PRP-gel preparations: fibrinet, RegenPRPKit, Plateltex and one manual procedure. Vox Sang. 2009;97:110–8. 19392780 10.1111/j.1423-0410.2009.01188.x

[11] van der Bijl I Vlig M Middelkoop E. Allogeneic platelet-rich plasma (PRP) is superior to platelets or plasma alone in stimulating fibroblast proliferation and migration, angiogenesis, and chemotaxis as relevant processes for wound healing. Transfusion. 2019;59:3492–500. 31568583 10.1111/trf.15535

[12] Iervolino V Di Costanzo G Azzaro R. Platelet gel in cutaneous radiation dermatitis. Support Care Cancer. 2013;21:287–93. 23150187 10.1007/s00520-012-1635-0

[13] Piccin A Di Pierro AM Corvetta D. Severe skin radiodermatitis fully healed with the use of platelet gel and a hyperbaric chamber. Blood Transfus. 2016;14:552–4. 26674818 10.2450/2015.0191-15 PMC5111383

[14] Fioramonti P Fino P Ferrazza G. Platelet-rich plasma (PRP) in a post radiotherapy sternal ulcer in a patient with Hodgkin’s lymphoma. J Blood Disord Transfus. 2015;6.

[15] Di Costanzo G Loquercio G Marcacci G. Use of allogeneic platelet gel in the management of chemotherapy extravasation injuries: a case report. Onco Targets Ther. 2015;8:401–4. 25709472 10.2147/OTT.S68469 PMC4332310

[16] Piccin A Primerano M Di Pierro AM. Healing of a soft tissue wound of the neck and jaw osteoradionecrosis using platelet gel. Regen Med. 2016;11:459–63. 27346565 10.2217/rme-2016-0031

[17] Picardi A Lanti A Cudillo L. Platelet gel for treatment of mucocutaneous lesions related to graft-versus-host disease after allogeneic hematopoietic stem cell transplant. Transfusion. 2010;50:501–6. 19821947 10.1111/j.1537-2995.2009.02439.x

[18] Merolle L Iotti B Berni P. Platelet-rich plasma lysate for treatment of eye surface diseases. J Vis Exp. 2022;186:e63772. 10.3791/63772 35993713

[19] Cox JD Stetz J Pajak TF. Toxicity criteria of the radiation therapy oncology group (RTOG) and the European organization for research and treatment of cancer (EORTC). Int J Radiat Oncol Biol Phys. 1995;31:1341–6. 7713792 10.1016/0360-3016(95)00060-C

[20] National Cancer Institute, Division of Cancer. Treatment and diagnosis cancer therapy evaluation program. common terminology criteria for adverse events v4.0. Available at: https://ctep.cancer.gov/protocolDevelopment/electronic_applications/docs/CTCAE_4.03.xlsx.

[21] Luzo ACM Fávaro WJ Seabra AB. What is the potential use of platelet-rich-plasma (PRP) in cancer treatment? A mini review. Heliyon. 2020;6:e03660. 32258495 10.1016/j.heliyon.2020.e03660 PMC7113436

